# Sustainable Green Synthesis of Yttrium Oxide (Y_2_O_3_) Nanoparticles Using *Lantana camara* Leaf Extracts: Physicochemical Characterization, Photocatalytic Degradation, Antibacterial, and Anticancer Potency

**DOI:** 10.3390/nano12142393

**Published:** 2022-07-13

**Authors:** Rajakumar Govindasamy, Mydhili Govindarasu, Salman S. Alharthi, Preeyanghaa Mani, Neppolian Bernaurdshaw, Thandapani Gomathi, Mohammad Azam Ansari, Mohammad N. Alomary, Banan Atwah, M. Shaheer Malik, V. Devi Rajeswari, Kaliaperumal Rekha, Saleh A. Ahmed, Muthu Thiruvengadam

**Affiliations:** 1 Collaborative Innovation Center for Advanced Organic Chemical Materials Co-Constructed by the Province and Ministry, Ministry of Education Key Laboratory for the Synthesis and Application of Organic Functional Molecules and College of Chemistry and Chemical Engineering, Hubei University, Wuhan 430062, China; microlabsraj@gmail.com; 2 Molecular Oncology Laboratory, Department of Biochemistry, Periyar University, Salem 636011, Tamil Nadu, India; mydhilibc12@gmail.com; 3 Department of Chemistry, College of Science, Taif University, P.O. Box 110999, Taif 21944, Saudi Arabia; s.a.alharthi@tu.edu.sa; 4 Nanotechnology & SRM Research Institute, SRM Institute of Science and Technology, Kattankulathur, Chennai 603203, India; mspreeyanghaa@gmail.com (P.M.); neppolian.b@res.srmuniv.ac.in (N.B.); 5 Department of Chemistry, D.K.M. College for Women, Vellore 632001, Tamil Nadu, India; chemist.goms@gmail.com; 6 Department of Epidemic Disease Research, Institute for Research and Medical Consultations (IRMC), Imam Abdulrahman Bin Faisal University, Dammam 31441, Saudi Arabia; maansari@iau.edu.sa; 7 National Centre for Biotechnology, King Abdulaziz City for Science and Technology (KACST), P.O. Box 6086, Riyadh 11442, Saudi Arabia; malomary@kacst.edu.sa; 8 Laboratory Medicine Department, Faculty of Applied Medical Sciences, Umm Al-Qura University, Makkah P.O. Box 7607, Saudi Arabia; baatwah@uqu.edu.sa; 9 Department of Chemistry, Faculty of Applied Sciences, Umm Al-Qura University, Makkah 21955, Saudi Arabia; mdshaheermalik@gmail.com; 10 Department of Biomedical Sciences, School of Biosciences and Technology, VIT, Vellore 632014, Tamil Nadu, India; sdevirajeswari@gmail.com; 11 Department of Environmental and Herbal Science, Tamil University, Thanjavur 613005, Tamil Nadu, India; lakhith14@gmail.com; 12 Department of Chemistry, Faculty of Science, Assiut University, Assiut 71516, Egypt; 13 Department of Applied Bioscience, School of Life and Environmental Science, Konkuk University, Seoul 05029, Korea; 14 Department of Microbiology, Saveetha Dental College, Saveetha Institute of Medical and Technical Sciences (SIMATS), Chennai 600077, India

**Keywords:** *Lantana camara*, Y_2_O_3_ nanoparticles, photocatalytic degradation, cervical cancer, HeLa cells

## Abstract

Due to their appropriate physicochemical properties, nanoparticles are used in nanomedicine to develop drug delivery systems for anticancer therapy. In biomedical applications, metal oxide nanoparticles are used as powerful and flexible multipurpose agents. This work described a green synthesis of Y_2_O_3_ nanoparticles (NPs) using the sol-gel technique with the use of aqueous leaf extracts of *Lantana camara* L (LC). These nanoparticles were characterized with the aid of different methods, including UV, X-ray diffraction (XRD), Fourier transformed infrared spectroscopy (FTIR), transmitted electron microscopy (TEM), and photocatalytic degradation. Y_2_O_3_ nanoparticles showed excellent antibacterial activity against Gram-positive *Bacillus subtilis* and Gram-negative *Escherichia coli* with a 10 to 15 mm inhibitory zone. Green Y_2_O_3_ NPs were released with a 4 h lag time and 80% sustained release rate, indicating that they could be used in drug delivery. In addition, the bioavailability of green Y_2_O_3_ NPs was investigated using cell viability in cervical cancer cell lines. These green-synthesized Y_2_O_3_ NPs demonstrated photocatalytic degradation, antibacterial, and anticancer properties.

## 1. Introduction

Cervical cancer is the 4th majorly diagnosed cancer in women worldwide, with approximately 604,000 new cases and 342,000 global mortalities in 2020 [1,2,3,4]. Although the incidence of cervical cancer decreased over the last few decades, the trend in young women has been primarily influenced by a distant stage and cervical carcinoma [5,6,7]. Advanced cervical carcinoma is the most aggressive type of ovarian cancer, occurring in around 70% of cases in both developed and developing countries [8]. The major causes of cervical cancer are hormonal changes, obesity, overweight, weakened immune system, human papilloma viral infection, talcum powder, family history, and age, as identified by the International Cancer Research Agency [9]. Various methods, such as surgical therapy, chemotherapy, and radiotherapy, are used to treat cervical cancer [10,11]. Nanostructure-based diagnostics and treatment might be capable of overcoming the limitations of traditional methodologies, while simultaneously improving chemotherapeutic drug efficiency. Nanoparticles (NPs) are less than 100 nm in size and are used to shuttle drugs with specific target particles. These NPs are targeted at specific infected sites to deliver effectively targeted cancer treatments [12,13,14].

The most critical components in nanotechnology research are metal oxide nanomaterials. They are significant because of their catalytic, magnetic, electrical, and mechanical properties. Oxidative nanostructures are favorable in a wide range of thermal, photonic, mechanical, power, electrostatic, physicochemical, and gas-sensitive applications due to their enormous surface-to-volume ratios [15,16,17]. The development of profitable and low-cost ways to enhance the efficacy of anticancer therapy is urgently needed. Over the past 20 years, significant progress in nanotechnology and nanoscience has been achieved, providing us with a new perspective to overcome the drawbacks of conventional cancer therapy [18,19]. Metal oxide nanoparticles are used as powerful and flexible multipurpose agents [20]. Diverse metal oxides and hydroxide-derived nanomaterials, such as zinc oxide, copper oxide, Europium (III) hydroxide [21], and iron oxide nanoparticles [22], have been used in the production of bioengineering scaffolds [23,24].

Green synthesis is considered the most economical, sustainable, efficient, and reliable approach to nanoparticle synthesis [25,26]. This technique does not require toxic substances, high temperatures, or high pressure and does not adversely affect public health and the environment [27]. Green chemistry, which uses plant biomass or plant minerals, has become a substitute for nanoparticle non-toxic, eco-friendly, and sustainable production [28]. The development of nanomaterials thus plays a vital role in improving anti-inflammatory features with low toxicity [29]. Plants are a great source of bioactive metabolites, such as pentacyclic triterpenoids, phytosterols, polyphenols, steroids, saponins, iridoid glycosides, oligosaccharides, tannins, phenolic acids, phycobatannin, anthocyanins, and proanthocyanidins [30]. Using bioactive phytochemical compounds, scientists investigated plant chemistry, toxicology, and pharmacology [31].

*Lantana camara* L., commonly called “Unnichedi” (Tamil) and “pulikampa” (Telugu), is a species of flowering plant of the Verbenaceae family that is widely recognized as a significant weed in India. *Lantana camara* (LC) is an important plant containing alkaloids, terpenoids, phenolics, flavonoids, and other phytochemicals. *L. camara* is often used to treat various diseases in traditional medicine, including paludism, cancer, hypertension, tetanus, neoplasm, dermatitis, cut-offs, catarrh, abdominal viscera, measles, chickenpox, bronchitis, and fever. *L. camara* also has antifungal, anti-proliferative, antibacterial, nematicidal, germicidal, anti-ulcerogenic [32,33], antidiabetic [34], immunosuppressive [35], anthelminthic, anti-protozoal, antimicrobial [36], anti-inflammatory, and antipyretic activity [37].

Yttrium oxide (Y_2_O_3_), a widespread rare earth metal, is significant for future use due to its thermal stability and chemical and mechanical reliability. Yttrium oxide is used in biomedical images, materials science, synthesis of inorganic compounds, optics, electricity, biology applications [38,39], and photodynamic therapy. Furthermore, yttrium (Yb) and the widely doped rare earth metals erbium (Er) and europium (Eu) are non-cytotoxic when utilized in vivo. In this respect, Y_2_O_3_ nanoparticles of different sizes and morphologies were developed through various methods, such as sonochemical, solvothermal, hydrothermal, electrochemical, sol-gel, and thermal decomposition [16,40,41,42]. However, most of these approaches include costly and toxic chemicals as stabilizing or capping agents; their environmental applications are limited. Interestingly, a promising method to overcome these limitations was developed that involves the utilization of plant extracts.

In recent years, Y_2_O_3_ has been considered a crucial component of rare-earth substances and an excellent potential component in manufacturing optoelectronic equipment and chemical catalysis. The dielectric constant of Y_2_O_3_ is high, and the material has high thermal stability [43,44]. It can be used as a highly efficient stabilizer and functional composite materials, such as yttria-stabilized zirconia [45]. It is also widely employed as a host for rare-earth doping and is interested in potential biological and photodynamic image processing applications. Y_2_O_3_ NPs reduce oxidative stress-induced apoptosis and pancreas damage caused by diazinon in the rat pancreatic islets [46,47]. Plant metabolites can reduce cap metal ions in nanoparticles and contribute to their absorption [48]. This intrinsic ability arises from phytochemicals that could be suppressed with reduction and capping agents [49]. Therefore, *L. camara* leaf extract could meet the growing need for an alternative method of environment-friendly and economical synthesis of Y_2_O_3_ nanoparticles.

Photodegradation is a promising alternative because it produces hydroxyl free radicals, which can degrade many dyes [50,51,52]. The Y_2_O_3_ NPs are excellent substrates for rare earth metals and have strong luminescence effectiveness, and they could be utilized in photocatalytic therapies and biomedical diagnostics [53,54]. This study aimed to develop a new strategy for the green synthesis of Y_2_O_3_ NPs using *L. camara* aqueous leaf extract. Then, characterization of the products was carried out to see cell viability, biocatalytic, antibacterial, and anticancer activity in human cervical cancer HeLa cells.

## 2. Materials and Methods

### 2.1. Chemicals and Reagents

The following chemicals and reagents were purchased from commercial sources and used as received without further purification: Yttrium (III) acetate hydrate 99.0%, oleylamine of the technical grade 70%, ammonia hydroxide of reagent grade 30%, yttrium (III) nitrate of the hexahydrate 99.8%, anhydrous of the reagent grade of the ammonium hydroxide 99.5%, chloral hydrate 99.8%, *n*-hexane, gelatine solution, sodium hydroxide, Dulbecco modified eagle media (DMEM), fetal bovine serum (FBS), and MacConkey agar medium.

### 2.2. Preparation of Leaf Extracts

*Lantana camara* L. fresh leaves were collected and washed with tap water and then washed with double distilled water until no impurities remained. The *L. camara* leaves were dried in the shade for ten days to remove residual moisture. The dried leaves were pulverized in a sterile electric blender to obtain a fine powder and stored in an airtight bottle avoiding sunlight for further use. Then, 10 g of leaf powder was mixed thoroughly with 200 mL of double distilled water and heated for 10 min at 60 °C. This was followed by cooling and filtration through Whatman No.1 filter paper to afford the leaf extract [55,56]. The filtered extract was collected and kept for further studies.

### 2.3. Y_2_O_3_ Nanoparticles Synthesis and Characterization

Nanoparticles of Y_2_O_3_ were synthesized in the presence of gelatin through precipitation with ammonium hydroxide. In this method, 75 mL of 0.1 M yttrium nitrate hexahydrate (Y(NO_3_)_3_·6H_2_O) aqueous solution was added to 100 mL of ammonium hydroxide, and the solutions were mixed and stirred by REMI Electromagnetic stirrer (Chennai, Tamil Nadu, India). The precipitate formed was kept for 4 h at room temperature, washed several times with de-ionized water, centrifuged at 8000 rpm, and finally resuspended in ethanol. The obtained slurry was dried in a hot air oven for 24 h at 70 °C and pulverized with mortar and pestle. The powder was calcined to crystal-clear Y_2_O_3_ nanocrystals at 650 °C for four h [16,57]. The UV–vis spectrum was recorded with a spectrophotometer (Shimadzu UV 1800, Torrance, CA, USA) at 37 °C. XRD characterized the LC Y_2_O_3_ nanoparticles on a Bruker D8 automated multipurpose powder X-Ray Diffractometer with Cu-K radiation of wavelength 1.5406 nm. The Infrared Spectra of the LC Y_2_O_3_ NPs were obtained using an FTIR-spectrophotometer (Therma Nicolet Corp, MA, USA). In contrast, a field emission gun was used for TEM analysis at a 200 kV accelerating voltage in a FETEM apparatus (JEOL FETEM, 200 kV, Tokyo, Japan) [58].

### 2.4. Photocatalytic Evaluation

The photo-degradation of Rhodamine B (RhB) revealed the photoelectrocatalytic activity of the LC Y_2_O_3_ NPs. The photodegradation was examined by a 250W Xenon lamp with high pressure, with the liquid approximately 10 cm from the Xenon bulb illumination. In a traditional photodegradation procedure, 20 mg of LC Y_2_O_3_ NPs were added to a 20 mL aqueous solution of 10 µM RhB (10 ppm). To obtain dye adsorption-desorption equilibrium on the catalyst, the solution was stirred for 120 min in the dark before being subjected to visible light for different time intervals. Approximately 4mL of the solution was taken every 30 min throughout photocatalytic degradation and centrifuged at 750 rpm for 5 min to remove the excess catalytic nanoparticles. Subsequently, the amount of RhB was instantly observed using a UV-Vis spectrophotometer (Shimadzu UV 1800, Torrance, CA, USA) to determine the absorption in the spectral region of 500–750 nm [59]. Under the same conditions, the spontaneous photocatalytic degradation of RhB (i.e., photolysis) was investigated without a photocatalyst [60]. The following formula can be used to determine the photocatalytic degradation rate of the catalyst.

(1) $$Degradation\;efficiency\;\left(\%\right)=\frac{\left(Ci-Cf\right)}{Ci}\times 100$$

where *Ci* represents the initial concentration of RhB, and *Cf* represents the final concentration of the dye after a specified reaction time (min).

### 2.5. Antibacterial Activity

The agar diffusion method was used in the microbiological experiment [46]. The antibacterial effect of the LC Y_2_O_3_ NPs was examined using microbes such as *E. coli* (MTCC 732) and *Bacillus subtilis* (MTCC 5981). The bacterial strain was acquired from the MTCC (Microbial-Type-Culture-Collection) at IMTECH in Chandigarh, India. The microbiological suspension culture was maintained for 24 h in nutrient broth; the prepared MacConkey agar medium was heated at 120 °C for 15 min, and 20 mL of sterile agar medium was poured into Petri plates. The bacterial culture was spread uniformly over agar plates using a cotton swab stick. Following inoculation, various concentrations (50, 100, 150, and 200 µmol/L) of LC Y_2_O_3_ NPs dissolved in DMSO and sonicated for 15 min were placed onto the sterile discs (6 mm). The microbes were then inoculated into discs or plates and kept at 37 °C for 24 h for bacterial species with a triplicate test performed for each microbe. The size of the inhibition zone formed on each disc was determined (mm).

### 2.6. Cytotoxicity Assay

Human HeLa cell lines were obtained from the National Centre for Cell Science (NCCS) in Pune, India. DMEM was used to maintain the added cells with 10% FBS. In the median, penicillin (100 μg/mL) and streptomycin (100 μg/mL) were added to avoid bacterial contamination. The HeLa cells were positioned in 24 wells (1 × 10^5^ cells/well) and incubated in a 5% CO_2_ incubator at 37 °C. After the cells were placed in wells and kept in the incubator for 24 h, nanoparticles were added at concentrations of 1.56, 3.12, 6.25, 12.5, 25, 50, and 100 µg/mL. The samples were then removed from the well and washed with DMEM. 3-(4,5-Dimethyl-2-thiazolyl)-2,5-diphenyl tetrazolium-bromide (0.5% MTT reagent) was added (10 μL/well) to each well and incubated for 4 h. The spectrophotometer measured the optical density at 540 nm (Shimadzu UV 1800, Torrance, CA, USA) [59]. A nanoparticle’s half-maximal inhibitory concentration (IC50) measures whether it can prevent a specific biological or metabolic function [60]. The following formula was used to determine the percentage of inhibition of proliferation:

(2) $$Viability\;cells\;inhibition\;\left(\%\right)=100-\left[\frac{\left(A_{t}-A_{b}\right)}{\left(A_{c}-A_{b}\right)}\right]\times 100\%$$

*A_t_* = absorption of test compound, *A_b_* = absorption of blank, *A_c_* = absorption of control.

### 2.7. In Vitro Drug Release

The dialysis technology examined drug release rate from synthesized samples [60]. In brief, the dialysis tube was filled with 2 mL of the LC leaf extract, Y_2_O_3_ NPs, and LC Y_2_O_3_ NPs dispersion (≈10 mg of NPs) (12 kDa, Sigma, Aldrich, MA, USA). As recommended to test the dissolution of vagina products, the dialysis tube was placed in 15 mL SVF (pH < 4.2) and kept at 37 °C and 100 rpm. Then, 1 mL sample was removed from the medium at specific time intervals and replaced with a fresh medium (SVF: Stromvascular Fraction, pH ≈ 4.2) to provide the sample’s persistence. A UV-Visible spectrometer was used to measure the concentration in each sample at 298 nm (Shimadzu UV, 1800, Torrance, CA, USA).

## 3. Results

### 3.1. Characterization of LC Y_2_O_3_ NPs

**Figure 1:**
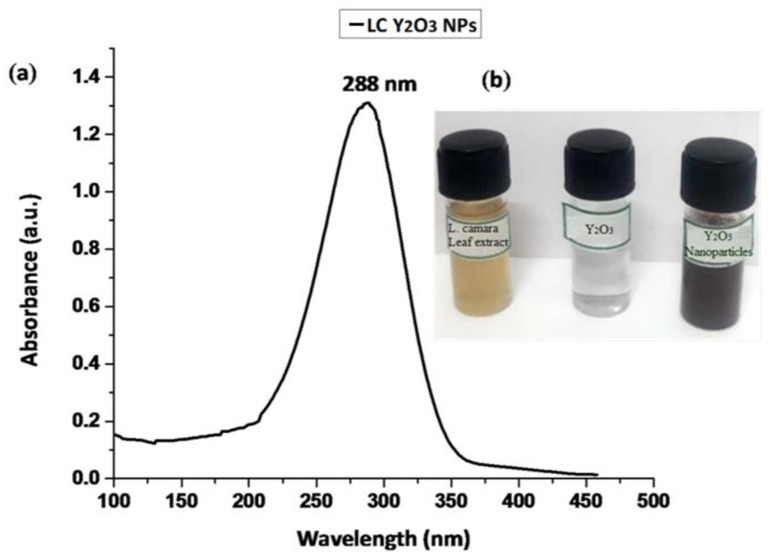
UV-vis absorption spectra of (**a**) green synthesized LC Y_2_O_3_ NPs suspended in ethanol and (**b**) the color changes following the synthesis of LC Y_2_O_3_ NPs.

**Figure 2:**
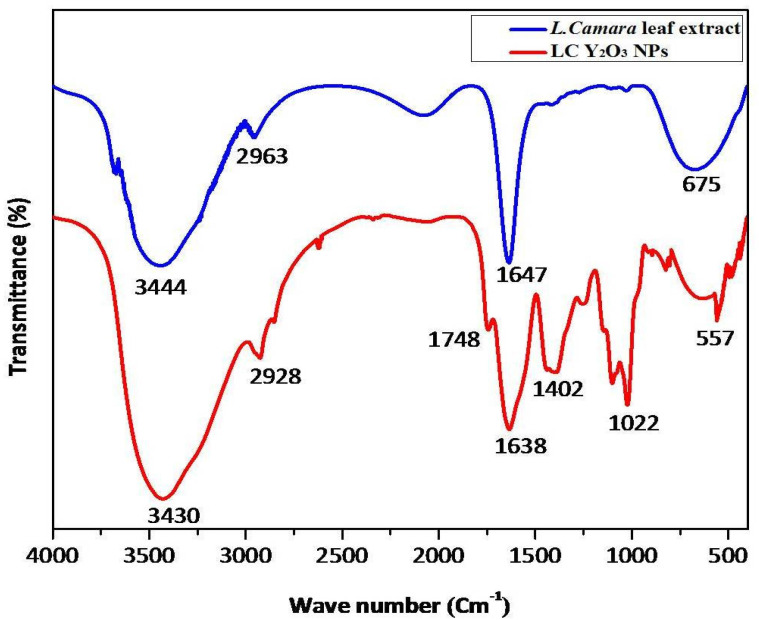
FTIR spectrum of the *L. camara* leaf extract and green-synthesized LC Y_2_O_3_ NPs.

The effect of green synthesized LC Y_2_O_3_ NPs was studied using UV-Visible spectroscopy. As demonstrated in Figure 1, the LC Y_2_O_3_-loaded NPs exhibited broad UV absorption with enhanced intensity at 288 nm. Similar results were reported for rare earth oxides [61] and organic semiconductor-coated silver particles [62], where such effect was attributed to absorption-induced scattering. The presence of multifunctional groups in the *L. camara* leaf extract and green synthesized LC Y_2_O_3_ NPs were investigated using FTIR. The infrared spectrum of *L. camara* leaf extract illustrates vibrational bands at 3444, 2963, 1647, and 675 cm^−1^. The absorption peaks of *L. camara* leaf extract at 3444 and 2963 cm^−1^ were attributed to O–H stretching and C–H antisym and sym stretching vibrations, respectively. The absorption peaks of *L. camara* leaf extract at 1647 and 675 cm^−1^ bands may be assigned to C=O in ketone esters and C–C–CHO bending in aldehydes, respectively. The absorption band at 1050 cm^−1^ was obtained by fabricating the green synthesized Y_2_O_3_ NPs [63,64,65], whereas the very wide band at 3425 cm^−1^ is attributed to the C–H stretching of H–bonded alcohols and phenols [66,67]. Similar results reported that the 600–400 cm^−1^ band is assigned to the M–O region (Y–O), whereas the 617 cm^−1^ band confirms the YO NPs’ extending vibration [68]. FTIR results of green synthesized LC Y_2_O_3_ NPs revealed 3430 cm^−1^ is assigned to the NH stretch with primary amines and 2928 cm^−1^ to C–H antisym and sym stretching vibrations. On the other hand, absorption bands at 1748 cm^−1^ and 1638 cm^−1^ are attributed to the C=O in esters and C=O stretch secondary amides, respectively. FTIR spectrum of green synthesized LC Y_2_O_3_ NPs showed bands at 1402 cm^−1^ due to O–H in carboxylic acids, 1022 cm^−1^ to CH–OH in cyclic alcohols with C=O stretch vibrations. Furthermore, the FTIR peaks observed at 557 cm^−1^ were ascribed to Y–O band stretching (Figure 2). The Y–O band (557 cm^−1^) indicates that the Yttria phase was formed at 600 °C [69].

**Figure 3:**
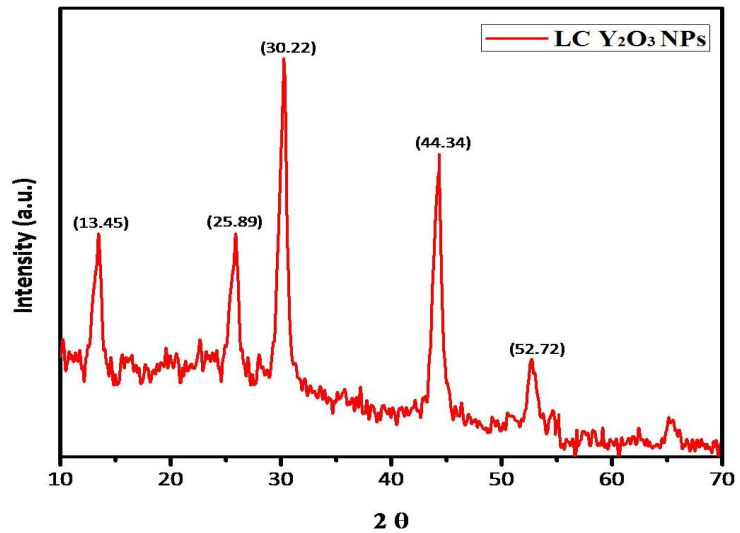
XRD pattern of green synthesized LC Y_2_O_3_ NPs (JCPDS card no. 83-0927).

The 2θ values of the green synthesized XRD detected Y_2_O_3_ NPs. Indeed, diffraction peaks of LC Y_2_O_3_ NPs at 2θ values of 13.45°, 25.89°, 30.22°, 44.34°, and 52.72° were indexed as 202, 211, 222, 431, and 440 (JCPDS card no. 83-0927) [70]. The phase evaluation of all synthesized samples was performed with XRD. Figure 3 provides XRD patterns of powders calcinated at different temperatures. In various crystalline phases, Y_2_O_3_ NPs could be found as polymorphs. For all samples, the XRD analysis confirms the formation of a single-phase orthorhombic Y_2_O_3_ structure, revealing the successful synthesis of Y_2_O_3_ NPs. According to the method of Hanawalt (222) and (431), the cubic Y_2_O_3_ stage was characterized by sharp peak diffraction angles of 30.22° and 44.34°, respectively [71].

**Figure 4:**
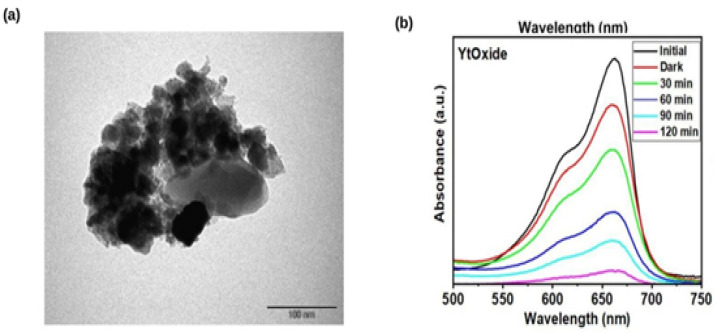
(**a**) TEM image of the green synthesized LC Y_2_O_3_ NPs. (**b**) Photocatalytic degradation of Rhodamine B utilizing the green synthesized LC Y_2_O_3_ NPs.

Meanwhile, TEM images show these particles’ tendency to form agglomerates ranging from 20 nm to 45 nm. The Y_2_O_3_ NPs display high crystallinity in the TEM images in Figure 4a. Moreover, the coating layer of Y_2_O_3_ is very thin in the composite samples, which agrees with the lack of diffraction peaks related to the formation of Y_2_O_3_ particles in Figure 4a. The images show crystalline particles with nearly orthorhombic structure and some hexagonal particles. Figure 4a shows a TEM image of green synthesized LC Y_2_O_3_ NPs with irregular shapes. The Scherrer equation estimated the crystallite size of green synthesized LC Y_2_O_3_ NPs at 30 nm.

### 3.2. Photocatalytic Activity of Green Synthesized LC Y_2_O_3_ NPs

The photodegradation of dyes without a catalyst was evaluated by exposing the blank dye to illumination. For 120 min, absorbance measurements on blank dye were conducted at different time intervals. The degradation of Rhodamine B dye (RhB) was used to illustrate the photocatalytic efficiency of LC Y_2_O_3_ NPs. In the absence of the catalyst, only 18% of the dye degraded after 60 min of exposure, indicating that the catalyst played a significant role in dye photocatalytic degradation [67]. A reduction in peak intensity over time indicates that the dye concentration was considerably reduced and that the catalyst was photoactive. The efficiency of LC Y_2_O_3_ NPs as a photocatalyst was investigated using LC Y_2_O_3_ NPs for photocatalytic degradation of Rhodamine B. Based on these findings, 92% of the dye was degraded within 60 min, as depicted in Figure 4b. When photons with higher intensity than the bandgap energy of LC Y_2_O_3_ NPs were absorbed, electrons were excited to the conduction band, causing a vacancy in the valence band. The electrons in conduction produce (O_2_^−^•) free-radicals by transferring an electron to the oxygen molecule. In contrast, the vacancy in the valence band generates hydroxyl radicals (•OH), which react with water molecules [72]. These produced reactive oxygen species (ROS), which destroyed the Rhodamine B dye. The reactions result in dye decomposition in the presence of eco-friendly synthesized LC Y_2_O_3_ NPs.

### 3.3. Green-Synthesized LC Y_2_O_3_ NPs Antibacterial Activity

**Figure 5:**
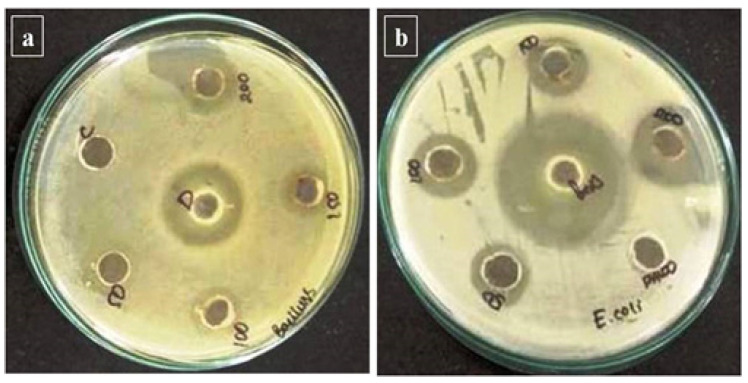
The green synthesized LC Y_2_O_3_ NPs showed an improved zone of inhibition against (**a**) *Bacillus*, and (**b**) *E. coli*, compared to Y_2_O_3_ NPs.

The antibacterial activity of green-synthesized Y_2_O_3_ NPs produced in LC aqueous leaf extracts (50–200 µL) was studied against gram-positive and negative bacterial strains. The green synthesized LC Y_2_O_3_ NPs with typical antibacterial activity (the zone of inhibition) ranged from 10 to 15 mm for the selected bacterial strains. *Bacillus* was found to have the highest activity (15 mm inhibition). The diameter of the zone of inhibition was 15 and 11 for *Bacillus* and *E. coli* (11mm), respectively (Figure 5a,b). Compared with the antibiotics and LC Y_2_O_3_ NPs showed an excellent inhibition zone. In leaf synthesized Y_2_O_3_ NPs, there was a slight increase in the inhibition zone compared to the control. The results show that the new LC Y_2_O_3_ NPs are promising antimicrobials for plant pathogens. Previously reported Y_2_O_3_ NPs were effective against *E. coli*, *P. aeruginosa*, *S. marcens*, and *S. aureus* [73].

### 3.4. Cytotoxicity of Y_2_O_3_ NPs in HeLa Cell Lines

**Figure 6:**
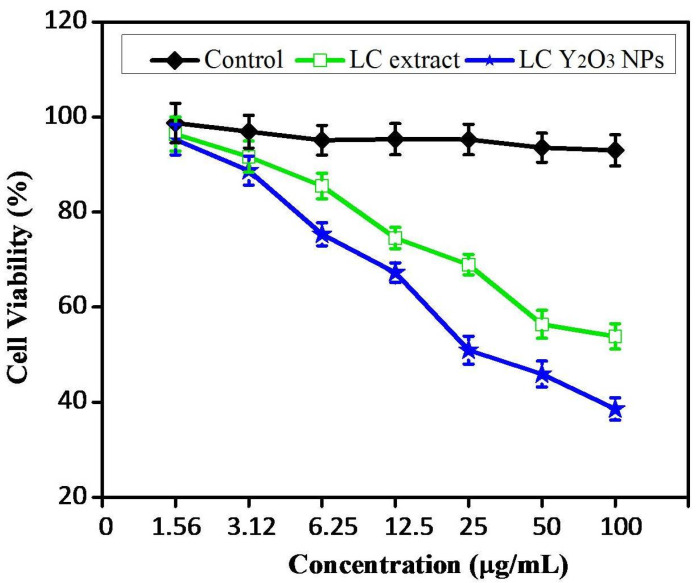
Green LC Y_2_O_3_ NPs inhibit the cell viability of human cervical carcinoma HeLa cells. HeLa cells were treated for 24 h with various concentrations of LC extract and green LC Y_2_O_3_ NPs.

The MTT assay evaluated the cytotoxicity of different doses of synthesized leaf extract and LC Y_2_O_3_ NPs (Figure 6). These results showed that treating cells with 25 µg/mL of synthetic Y_2_O_3_ NPs reduces cell viability by 52.6% compared to the control and reduces significantly to around 40.2% (Figure 6). The treatment with 50 μg/mL of green synthesized LC Y_2_O_3_ NPs decreased the cell viability by about 59%, and treatment with 100 µg/mL of Y_2_O_3_ NPs reduced the cell viability by around 32%.

Y_2_O_3_ NPs are particularly important for medical applications because they are considered drug-supply candidates. The major mechanism is Y_2_O_3_ NPs penetrating the cell wall and causing cell breathing distress. In the microorganism’s cell wall, Y_2_O_3_ NPs also penetrate the cell and destroy it by combining the action of sulfur and phosphorus compounds, such as proteins and deoxyribonucleic acid. Moreover, different concentrations (0, 1.56, 3.12, 6.25, 12.5, 25, 50, and 100 μg/mL) of NPs were used to treat the HeLa cells at 24 h. The results showed time- and dose-dependent declines in cell viability decreased by 53 to 98% in the 24 h treatments. These findings suggest that cell viability decreases when the concentration of NPs increases. The green synthesized LC Y_2_O_3_ NPs IC50 value against HeLa cell lines was determined after 24 h and was found to be 25 µg/mL.

### 3.5. In Vitro Drug Release

**Figure 7:**
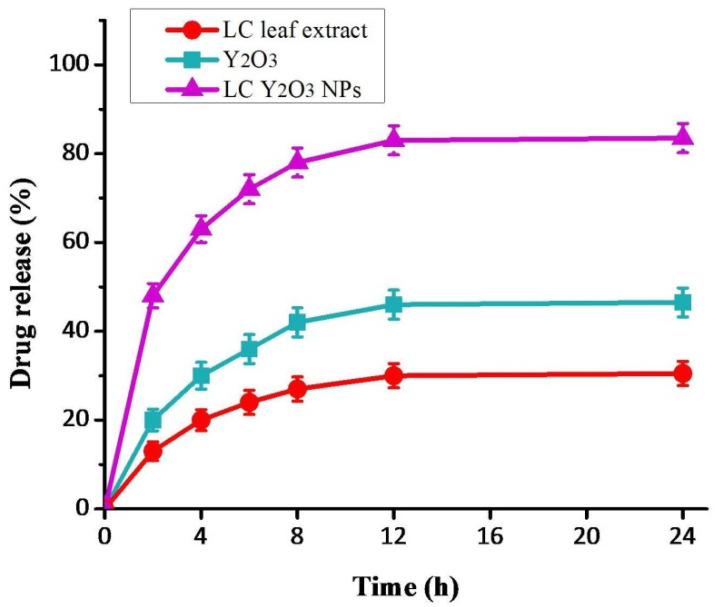
In vitro release of optimized LC Y_2_O_3_ NPs. The drug shows rapid NPs release and aqueous drug solution. Around 85% of the drug was released in the first 30 min of the study. A saturation status was then observed in the drug release. On the other hand, optimized LC Y_2_O_3_ NPs showed a more controlled drug release pattern than LC leaf extract and Y_2_O_3_ NPs.

Nanoparticles could be used as anticancer drugs in the treatment. They can penetrate cancer cells for targeted therapy by a smaller number of NPs. Large vascular pores deliver oxygen and nutrients to cancer sites and inflammatory tissues in these locations, allowing NPs to pass through and accumulate [73,74]. The curve would have an initial release, but the not whole drug was released before 4 h. The contrast to the behavior of this release can be explained by the reservoir function of the NPs, which release the drugs (Figure 7). Otherwise, the medicine is released immediately from NPs, as it is in the biochemical condition in these formulations [75]. This release pattern is advantageous for formulating various pharmaceutical drugs, especially where the preferential release rate is more desirable, such as in anticancer therapy.

## 4. Conclusions

In conclusion, we synthesized Y_2_O_3_ NPs from *L. camara* aqueous leaf extract with high surface reactivity and biocompatibility. These nanoparticles were characterized using various techniques, such as X-ray diffraction and FTIR, which revealed that they were pure single-phase and crystalline LC Y_2_O_3_ NPs with orthorhombic shapes. TEM analysis of green synthetic LC Y_2_O_3_ powder confirmed the production of nanoparticles with an average size of 30 nm. The zone of inhibition of LC Y_2_O_3_ NPs was found to be *Bacillus* (15 nm) and *E. coli* (11 mm). The results of this study revealed that the green synthesized LC Y_2_O_3_ NPs are effective at degrading dyes from water and preventing bacterial growth. Thus, these NPs possess potential in water filtration, coatings, and the food-manufacturing sector. The cytotoxicity effects of LC Y_2_O_3_ NPs nanoparticles against HeLa cell lines were dose-dependent and can be used to treat cervical cancer in humans. Due to their strong surface reactivity, these NPs could be employed in cancer therapy to deliver targeted drug loading. These characteristics make the green synthesized Y_2_O_3_ NPs an appropriate option for use in medicinal applications. Furthermore, detailed in vivo studies are needed to determine the safety and effectiveness of these green synthesized LC Y_2_O_3_ nanoparticles.

## Data Availability

Not applicable.
